# Eligibility criteria for the UK Winter Fuel Payment: are we targeting the right people?

**DOI:** 10.1136/jech-2025-224619

**Published:** 2025-12-25

**Authors:** Kai Wan, Jonathon Taylor, Marcos Quijal-Zamorano, Joan Ballester, Shakoor Hajat

**Affiliations:** 1 London School of Hygiene & Tropical Medicine, London, UK; 2 Department of Civil Engineering, Tampere University, Tampere, Pirkanmaa, Finland; 3 ISGlobal, Barcelona, Spain; 4 University of Bern Institute of Social and Preventive Medicine, Bern, BE, Switzerland; 5 University of Bern Oeschger Centre for Climate Change Research, Bern, BE, Switzerland

**Keywords:** POVERTY, ENVIRONMENTAL HEALTH, Health inequalities, HOUSING, PUBLIC HEALTH

## Abstract

**Background:**

Cold weather remains a serious health threat in the UK and elsewhere, particularly for older adults. The Winter Fuel Payment has been a key government strategy to mitigate health risks linked to cold homes in the UK, but recent policy shifts have raised questions about whether income-based eligibility criteria effectively identify those most at risk.

**Methods:**

We analysed cold-related mortality in adults aged ≥75 across 324 local authority districts in England (2007–2019) using distributed lag non-linear models in a spatial Bayesian framework. Multivariate meta-regression was used to evaluate modification of cold effects by deprivation, income-based pension credit uptake, home energy efficiency and fuel poverty.

**Results:**

Areas in the highest quartile of fuel poverty had significantly greater cold-related mortality risk than those in the lowest quartile, with a 15.3% versus 13.1% increase in mortality risk at the first compared with the 50th percentile of wintertime temperature, ie, an absolute difference of 2.2% (p<0.001). This effect was stronger than the corresponding differences for energy efficiency (1.7%, p=0.04), income as indicated by pension credit uptake (0.6%, p=0.39) and deprivation-based measures, for which differences were minimal. Overall, an estimated 17% of cold-related deaths among people aged ≥75 were attributable to fuel poverty.

**Conclusion:**

Fuel poverty, an indicator designed to capture both low-income and housing energy efficiency, is a stronger predictor of cold-related mortality than income (as indicated by pension credit update) or deprivation-based indicators alone. Winter energy support schemes should consider fuel poverty metrics in their targeting to more effectively reduce health risks associated with cold homes and improve equity.

## Introduction

 Individuals spend a large proportion of their time indoors—typically around 65%, rising to over 80% among older adults.^1^ Low indoor temperatures contribute to excess morbidity and mortality in the UK and other countries, particularly among older people and those with pre-existing health conditions.^2^ Previous research estimated 21.5% of excess winter deaths (the additional deaths occurring during winter months compared with other times of the year) from cardiovascular diseases are attributable to low indoor temperatures in England; however, this is a dated assessment based on a limited 1991 indoor air temperature dataset.^2 3^ Given the lack of recent evidence, and because indoor temperatures are more readily modifiable than outdoor exposures, researchers are calling for more widespread monitoring of indoor environmental monitoring, including thermal comfort.^4^

Several factors contribute to cold homes, including poor energy efficiency, low income, deprivation and high fuel prices.^5^ Individuals who cannot adequately heat their homes experience fuel poverty. A recent review found associations between fuel poverty and excess winter mortality, elevated communicable and non-communicable diseases as well as worsened mental health and well-being.^6^

Since 2019, fuel poverty in England has been measured by the government using the low-income low-energy efficiency (LILEE) metric. Under this metric, a household is considered fuel poor if residents are living in a property with low home energy efficiency and, after spending the required amount to heat their home, have a residual income below the official poverty line (60% of median income after deducting housing costs).^7^ Between 2011 and 2018, fuel poverty was measured using the low-income high-cost (LIHC) metric, which identified households with above-average required fuel costs and residual incomes below the poverty line.^8^

Addressing fuel poverty is a national policy priority. The UK government has responded with housing upgrades, energy price caps and targeted heating assistance such as the Winter Fuel Payment (WFP).^9^ Similar programmes to the WFP operate in many European countries, including France^10^ and Spain,^11^ relying on income-based eligibility criteria. Such payments have the potential to improve health outcomes of those most vulnerable.^12^

In the UK before 2024, the WFP was paid to all pensioners. In winter 2024/25, however, the UK government limited eligibility to pensioners who receive pension credit or certain other benefits. Pension credit is an income-related benefit for older adults on low incomes. However, uptake is incomplete—30–40% of eligible individuals (2018–2023) did not claim,^13 14^ and others just above the threshold may still face hardship or inadequate heating. As a result of the WFP eligibility change, over 10 million pensioners became ineligible. While this sought to improve targeting and reduce costs, critics warned it may increase the number of pensioners below the poverty line or worsen health impacts associated with cold homes.^15^ From winter 2025/26, the Government plans to restore eligibility to pensioners earning <£35 000. However, income-only criteria overlook critical housing-related vulnerabilities including energy inefficiencies and fuel poverty that significantly impact cold-related health outcomes.

## Methods

To inform whether WFP eligibility criteria are appropriate, we analysed the association between low outdoor temperatures in wintertime (Nov–Apr) and all-cause, cardiovascular and respiratory mortality (Office for National Statistics dataset) in all 326 English Local Authority Districts (LADs) apart from the Isles of Scilly and Isle of Wight, 2007–2019. The population of LADs in 2019 ranged from 9.7 thousand in the City of London to 1.1 million in Birmingham. The analysis focused on individuals aged 75+, the age group most vulnerable to cold-related mortality. Night-time mean temperatures (8pm–8am, from the European Centre for Medium-Range Weather Forecasts Reanalysis v5 (ERA5) – Land dataset)^16^ were used since people predominately stay home at night, making them the most relevant exposures for heating and fuel poverty impacts. Nevertheless, day and night-time temperatures are highly correlated (mean r=0.97), so results are likely robust to metric choices. Using distributed lag non-linear models in a spatial Bayesian framework—a novel spatial method adapted for small-area analysis^17^—we estimated the relative risk (RR) of mortality associated with extreme cold (first against 50th percentile of wintertime night-time temperature distribution, Nov-Apr) to represent cold-related mortality risk. Full details of the data and methods are provided in the online supplemental appendix.

**Figure 1:**
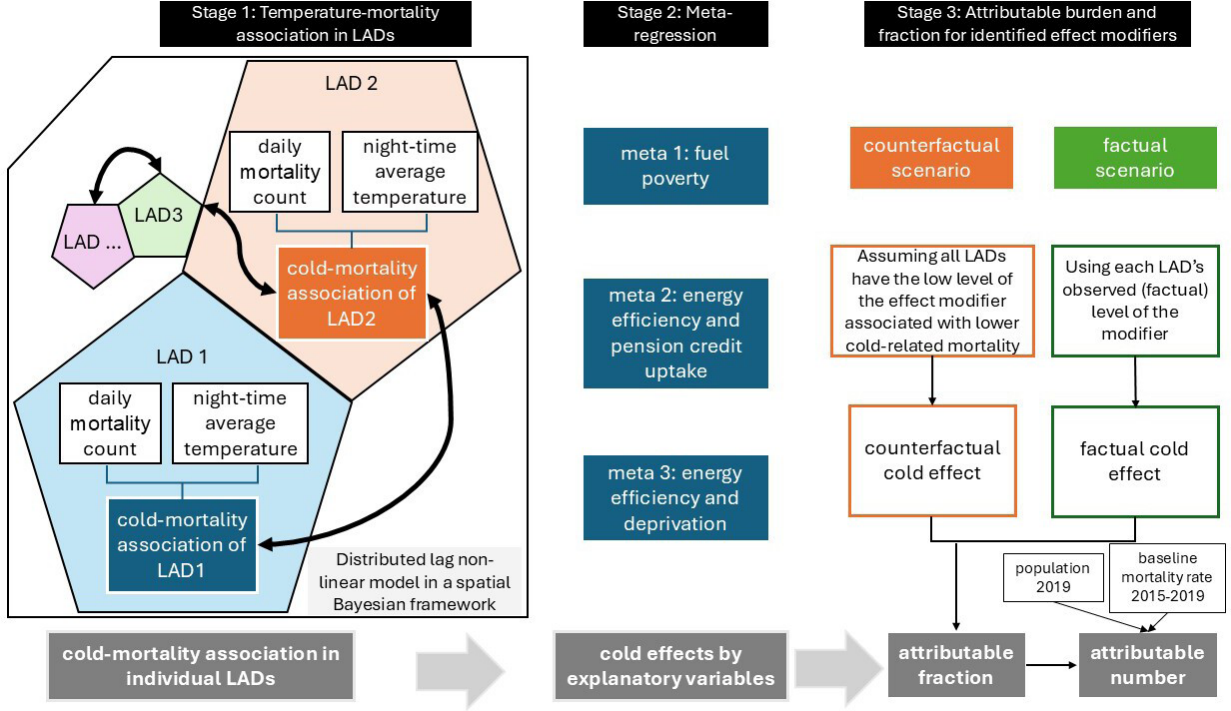
Conceptual diagram of the study methodology. The arrows in stage 1 indicate that the risk function for each location was fitted using data from the location itself, its adjacent locations and the overall pooled risk function for England. This fitting process is iterative for an optimal fit for all locations using a spatial Bayesian approach.

Using multivariate meta-regression, we examined how cold-related mortality risk varied by deprivation (Index of Multiple Deprivation, IMD), low income (indicated by pension credit uptake; see eligibilities in online supplemental appendix), energy inefficiency (Energy Performance Certificate rating of D or below) and fuel poverty (Department for Energy Security & Net Zero dataset).^18^ The LILEE (2019) was the main indicator of fuel poverty, with the LIHC (2018, see definitions in the Introduction) also considered. The analytical approach is summarised in figure 1. It consists of (1) a time-series regression analysis using spatial-Bayesian distributed lag non-linear models to derive the temperature-mortality association across the 324 LADs; (2) meta-regressions to examine the effects of fuel poverty, income (based on pension credit), deprivation and energy efficiency on cold-related mortality risk; and (3i) a health impact assessment to quantify the population attributable burden to identified effect modifiers. Further details of each stage of the analysis are provided in the online supplemental appendix.

## Results

**Figure 2:**
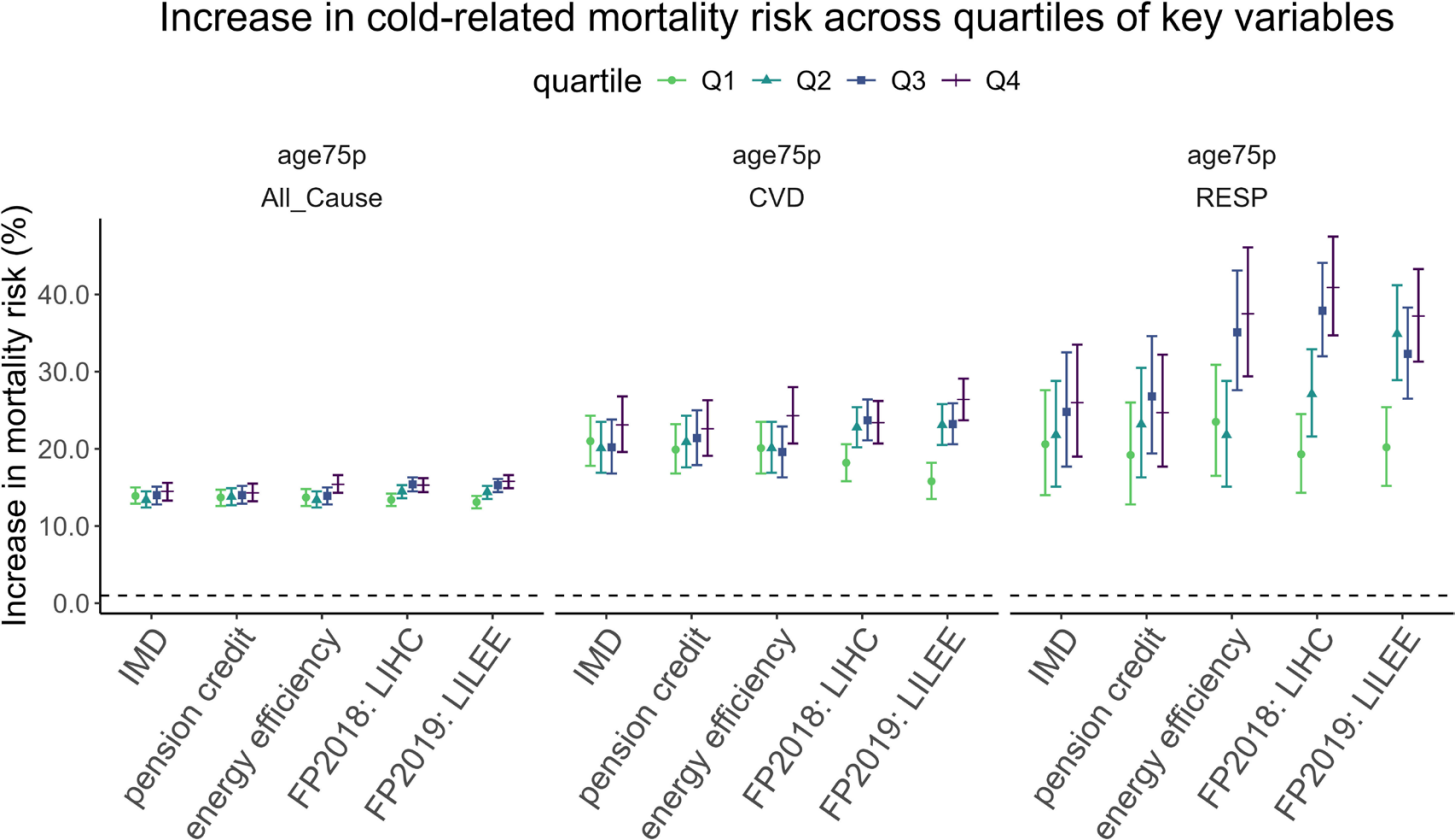
Increase in cold-related mortality risk at the 1st versus the 50th percentile of wintertime temperature (2007–2019), stratified by the Index of Multiple Deprivation (IMD), pension credit, energy efficiency and fuel poverty (FP) indicators—low-income low-energy efficiency (LILEE) measured in 2019 and low income high cost measured in 2018 (LIHC) in England. Mortality outcomes are presented for all-cause, cardiovascular (CVD) and respiratory (RESP) mortality in those aged 75 and over. For deprivation (IMD) and income (pension credit), energy efficiency was additionally controlled; for energy efficiency, deprivation was controlled. For fuel poverty models, income, deprivation and energy efficiency were not controlled to avoid adjusting away key components of the fuel poverty measurement. Location-specific random effects have been controlled in all models.

Cold-related mortality risk increased with both pension credit and fuel poverty, but the modification effect was strongest for fuel poverty (figure 2). The increase in cold-related mortality risk was greater in LADs with the highest quartile of pension credit recipients compared with those in the lowest quartile (14.3% vs 13.7%, a difference of 0.6%, p=0.39), and similarly higher in LADs with the highest quartile of energy-inefficient dwellings compared with the lowest quartile (15.4% vs 13.7%, difference: 1.7%, p=0.04). Notably, the modifying effect of fuel poverty (LILEE) on cold-related mortality risk was greater—15.3% increase in LADs with the highest fuel poverty versus 13.1% in the lowest (difference: 2.2%, p<0.001). This gap widened for cardiovascular (CVD) and respiratory (RESP) deaths—26.4% (CVD) and 37.2% (RESP) increase in cold-related mortality risk in LADs with the highest fuel poverty versus 15.8% (CVD) and 20.2% (RESP) in the lowest. Using these estimates, approximately 17% (empirical CI: 3.2% to 29.6%) of cold-related deaths from all causes among people aged 75+ were attributable to fuel poverty, accounting for 610 excess deaths annually (eCI: 580 to 642). The attributable fraction and annual burden for the other variables (ie, income, deprivation and energy efficiency) were not quantified because the differences in cold-related mortality risk between the highest and lowest quartiles of these measures were small and estimated with low precision, providing insufficient evidence of a meaningful modification effect. The descriptive statistics and exposure-response functions are included in the online supplemental appendix.

## Discussion

The findings support reconsideration of WFP eligibility. Targeting based on fuel poverty—particularly metrics that include housing energy efficiency—may better identify individuals at highest risk of adverse cold-related health effects compared with income-based criteria alone (indicated by pension credit update). Fuel poverty reflects a broader set of structural vulnerabilities, including income and housing thermal inefficiency. A policy framework integrating fuel poverty into eligibility may enhance equity and effectiveness.

However, several considerations must guide implementation. The LILEE metric captures energy efficiency but does not reflect energy prices. During price spikes, such as the energy crisis in 2022, it may underestimate need. Complementary indicators, such as the LIHC metric or self-reported difficulties with heating, may provide additional insight. When the previous Government changed its fuel poverty indicator from LIHC to LILEE in 2019, most (88%) of previously fuel poor households were still identified, but approximately 100 000 of those in energy efficient homes were excluded.^19^

Subjective measures (self-reported inability to heat) can also be used. However, overlap between subjective and objective definitions is low; 60% of self-identified fuel poor were not captured by LIHC.^20^ There is no single best fuel poverty indicator,^20^ and hence it is crucial to consider multiple indicators to improve health and equity. As the government has previously stated, when there was a change in the fuel poverty metric in 2019, those identified as fuel poor previously should still be considered in fuel poverty policies.^19^

Furthermore, not all individuals in fuel poverty live in cold homes, and vice versa. Behavioural factors, knowledge of heating systems and the dangers of cold exposure and social support also affect exposure. Nonetheless, our study reveals that fuel poverty remains a stronger proxy for cold-related risks than income alone (indicated by pension credit update). Additionally, in 2023, pension credit and fuel poverty prevalence among pensioners were similar (~12–13%), so shifting to fuel poverty-based targeting may have similar costs but better targeting.

Our analysis is based on population-level data at the LAD scale. While it reveals important trends, it cannot assign risk to individuals. The fuel poverty effect may be underestimated, as even areas with the lowest quartile of fuel-poor households contain households experiencing fuel poverty. For the same reason, the effects of income, deprivation and energy efficiency are also likely to be underestimated. Additionally, we used outdoor temperature measurements since indoor temperature data remain limited.

Despite these limitations, this analysis provides useful evidence that fuel poverty significantly drives cold-related mortality and is a useful policy guide. Although income remains important, it is insufficient on its own to capture vulnerability to cold-related health harms. In addition to the WFP, the UK also operates the warm home discount (WHD) offering £150 off energy bills to pensioners on pension credit and non-pensioner households on means-tested benefits. From 2022/23 to 2024/25, non-pensioner eligibility required both means-tested benefits and high energy costs,^21^ demonstrating the feasibility of incorporating energy costs into eligibility policy. However, it remains limited to those on means-tested benefits, whereas many ineligible households still struggle to heat homes. For example, Sweden and Slovenia—despite lower income inequality—show large well-being gaps between fuel-poor and other households, likely due to infrastructure and energy system factors.^22^

With policies such as the WFP and WHD evolving, there is a timely opportunity to reconsider eligibility criteria to ensure that they benefit those most in need and reduce health burdens of cold homes. We argue that incorporating fuel poverty into eligibility criteria could improve public health and reduce inequalities.

Understanding the health risks of fuel poverty and cold homes is important for medical and public health professionals in identifying at-risk patients, referring patients effectively and improving collaboration across sectors. Although this study was conducted for the UK, similar considerations could inform other countries, although location-specific research remains necessary for local context. As energy prices remain high and volatile and health inequalities deepen, this approach is both evidence-based and urgent.

## Supplementary material

10.1136/jech-2025-224619 online supplemental file 1
