# Supplementary material for: Eligibility criteria for the UK Winter Fuel Payment: are we targeting the right people?
Source: J Epidemiol Community Health. 2025 Dec 25;80(6):e224619. doi: 10.1136/jech-2025-224619 (PMC13217030; doi:10.1136/jech-2025-224619)
Supplement: online supplemental file 1 [file jech-80-6-s001.docx]

**Supplementary File – Detailed Methods**

This study aimed to estimate cold-related mortality risks and assess how indicators such as fuel poverty, energy efficiency, and deprivation and pension credit modify these risks across Local Authority Districts (LADs) in England. We also quantified the proportion of cold-related deaths attributable to fuel poverty among older adults (aged 75+).

# 1. Data

## 1.1. Mortality

Daily mortality counts in England in 2007-2019 for two age groups: age 0-74 and age 75+ and three groups of causes of death (CoD): all-cause, cardiovascular diseases (CVD; International Classification of Disease, ICD 10: I00-I99) and respiratory diseases (RESP; ICD10: J00-J99) in individual local authority districts (LAD) were obtained from the Office for National Statistics. More recent years were not considered due to the COVID-19 pandemic (Paniello-Castillo et al, 2025).

## 1.2. Population

Population size for two age groups (age0-74 and 75p) in 2019 were obtained at Lower Super Output Areas (LSOAs) level from the Office for National Statistics, which was then aggregated to LAD level to estimate the health burden.

## 1.3. Temperature

Hourly ambient temperatures at 9km grid spacing were obtained from the European Centre for Medium-Range Weather Forecasts (ECMWF) Reanalysis v5 (ERA5) – Land dataset. Daily night-time mean temperature in LADs was calculated by taking the average of the temperature between 8pm (the day before) and 8am of all grid points falling in an LAD. This resolution of this dataset is not enough to fully capture the Urban Heat Island effect. Nevertheless, the analysis of this study is on Local Authority District (LAD) level, so a 9km grid spacing is generally detailed enough to capture the average temperature in a LAD, apart from the very small LADs in London.

Exposure measurement error exists due to various factors such as incomplete representation of the Urban Heat Island effect, variation of temperature and population distribution within LADs—these all may affect the derived health risk function. However, this requires higher resolution temperature modelling for all of England and the entire period, which we do not have access to. We acknowledge this limitation and that there may be exposure measurement error in the temperature data.

## 1.4. Energy efficiency

Energy efficiency data were obtained from Energy Performance Certificates (EPC) on for individual domestic dwellings. Domestic EPCs have been issued when dwellings are constructed, sold or let since 2008 (Department for Levelling Up, 2024), if there has not been one in the past 10 years, meaning a dwelling can have multiple EPCs issued at different times. When more than one EPC is available for a dwelling, the latest one is selected for this study (up to Apr 2024). Additionally, this dataset does not hold data for every dwelling, and hence cannot be interpreted as a true representation of the whole of the housing stock in England and Wales. Additionally, care homes are not captured in domestic EPCs. The missing data was ignored in this study. Nevertheless, it is a useful open-source dataset providing detailed energy efficiency and housing characteristic information for a large number of households—a coverage of 60% (built before 1930) - 93% (after 2012) dwellings in England (ONS, 2024).

The incomplete coverage of EPCs may introduce some bias into the representativeness of the energy efficiency of LADs, as the availability of up-to-date EPC is lower for older homes, which are often less energy efficient. Therefore, the energy efficiency indicated by the EPC data is likely to be systematically higher than the real situation. However, due to the bias being largely systematic, as shown by a high correlation between the EPC coverage by dwelling type (ONS, 2023b), it has overall little impact when comparing the energy efficiency level across LADs. Nevertheless, more research is needed to take this bias into account.

The current household energy rating from A (highest energy performance) to G (least energy performance) rating (i.e. EPC rating) was extracted as an indicator of energy efficiency. It is based on the energy costs adjusted for floor area associated with space heating, water heating, ventilation and lighting, adjusting for savings from renewable energy generation technologies, such as micro-combined heat and power facilities and photovoltaics (Building Research Establishment, 2022). In addition to the EPC rating, the estimated household primary energy use in a 12 month-period per floor area was also extracted from the EPCs as another indicator of energy efficiency, and the correlation of these two variables is calculated to assess the consistency of the indices and the robustness in using EPC rating to reflect energy efficiency.

The original EPC data are provided at individual-dwelling level, which are aggregated into LAD levels by calculating the percentage of households with an EPC rating of D and below and the median energy consumption per floor area within a LAD.

## 1.5. Deprivation

The English Index of Multiple Deprivation (IMD) for 2019 was used to indicate deprivation of LADs. The IMD score was obtained for individual Lower Super Output Areas (LSOAs), and the average IMD score of LADs is calculated by weighing the population of LSOAs.

Deprivation from the Census 2011 was also obtained which contains four dimensions of deprivation: employment, education, health and disability, and household overcrowding (ONS, 2014). A household is classified as being deprived two or more of these dimensions are found in any combination in this study. The correlation of these two variables is calculated to assess the consistency of the deprivation indices and the robustness in using the IMD in indicating deprivation.

## 1.6. Pension credit

Pension Credit is an income-related benefit given by the government to certain pensioners e.g. low income, disabled, carer. Pension credit tops up the pensioner’s weekly income to £227.10 if they are single, or joint weekly income to £346.60 if they have a partner. Income includes State Pension, other pensions, earnings from employment and self-employment and most social security benefits, for example Carer’s Allowance. Not all benefits are counted as income. For example, the following are not counted: Adult Disability Payment, Attendance Allowance, Christmas Bonus, Child Benefit, Disability Living Allowance, Pension Age Disability Payment, Personal Independence Payment, social fund payments like Winter Fuel Payment, Housing Benefit and Council Tax Reduction. £10,000 or less in savings and investments do not affect Pension Credit, with every £500 over £10,000 counts as £1 income a week. See (GOV.UK, n.d.) for more details.

Quarterly pension credit caseload between May 2016 and Feb 2018 for LADs of England and Wales were provided by the Department for Work and Pensions and accessed through Stat-Xplore. Each case may be an individual or a couple if both are eligible for pension credit. We assume an average of 1.5 persons for each pension credit case to calculate the percentage of pensioners (aged between 65 and above in 2018) who receive pension credit in LADs.

## 1.7. Fuel poverty

Fuel poverty in England is currently measured by the Government using the Low-Income Low-Energy Efficiency (LILEE) metric. Under this metric, a household is considered to be fuel poor if they are living in a property with a fuel poverty energy efficiency rating of band D or below; and when they spend the required amount to heat their home, they are left with a residual income below the official poverty line (ONS, 2023). Subregional LSOA fuel poverty estimations (i.e. percentage of households in fuel poverty in individual LSOAs) using the LILEE metric are available from 2019 from the Department for Energy Security and Net Zero, which is aggragated to LAD level by weighing the number of households in LSOAs. In this study, the LILEE metric estimated in LADs for 2019 was used as the main indicator of fuel poverty.

Between 2011 and 2018, Fuel poverty in England was measured using the Low-Income High-Cost (LIHC) metric, where households are considered to be fuel poor if they have required fuel costs above average (the national median level); and were they to spend that amount, they would be left with a residual income below the official poverty line (Department for Business, 2020). Before 2011, the 10 percent metric is used to measure fuel poverty, where a household is fuel poor if it needs to spend more than 10% of its income on fuel to maintain an adequate level of warmth (usually 21 °C for the main living area, and 18 °C for other occupied rooms) (Department of Energy & Climate Change, 2013). The correlation between different fuel poverty measurements are explored.

## 1.8. Geographical unit and data linkage

The epidemiological analysis was conducted at LAD level using the 2016 boundary of the LADs. There are 326 LADs in England in 2016. This analysis unit was chosen for a balance between sufficient statistical power, granularity and computational demand. We attempted to conduct the analysis on postcode district level, whereas the computation was too demanding which cannot be finished within normal computation resource available. Mortality and temperature are matched by LAD and date. Other variables are matched LAD.

# 2. Epidemiological analysis

The epidemiological analysis was conducted through three stages. At stage 1, the association between ambient temperatures and daily mortality in individual LADs was analysed using a spatial Bayesian approach of distributed lag non-linear models (DLNM), which enables the characterisation of cold-related mortality risk in small areas by borrowing information from neighbouring areas (Quijal-Zamorano et al, 2024). At stage 2, the effect of deprivation, energy efficiency and fuel poverty on the cold-related mortality risk was assessed using random effects meta-regression. At stage 3, population attributable fraction of cold-related mortality due to fuel poverty was quantified.

## Stage 1: Temperature-mortality association in LADs

We employed a time-stratified case-crossover design in a Spatial-Bayesian framework to investigate the association between daily ambient night-time temperature and mortality. Only data between November and April is used for the analysis to focus on the effect of cold temperatures on mortality in the winter months when heating may be required. Analysis for the whole year (excluding the hottest three months) was not feasible given existing computing resources. Therefore, the analysis is restricted to November-April, which should have captured most of the cold-health impacts, similar to the month choices in previous research (Wan et al, 2022; Achebak et al, 2023).

Poisson regression was used to model the association between the daily mortality count and night-time temperature in individual LADs. To account for both non-linear exposure-response relationships and delayed effects of temperature on mortality, we incorporated distributed lag non-linear models (DLNMs) into the regression. The DLNM framework allows for simultaneous modeling of the non-linear exposure-response relationship and the lag-response relationship (Gasparrini et al, 2010). The association between temperature and mortality is modelled using a natural cubic spline with inner knots at the 30^th^ and 70^th^ percentile of LAD-specific winter night temperatures between 2007-2019. These location of these two knots are selected to capture the non-linearity around both the lower and higher end of the temperature range, where the non-linearity is more likely to occur, while also ensuring the knots are not too close to the extremes where estimates would be unstable.

The effects of cold temperature can persist for many days following exposure, i.e. the lagged effect. A previous study in England shows that the cold-related mortality risk from all-cause of deaths on individual lagged days peaked at a lag of 2-3 days, which gradually decreased and appears to be non-significant after 14 days (Hajat et al, 2016). Although there may be cold effect at longer lags, it’s likely that these represent more indirect cold effects (Arbuthnott et al, 2018). Therefore, a maximum lag of 14 days is considered in this research, and the association between lag and mortality is modelled using a natural cubic spline with 2 equally spaced inner knots at the log scale with an intercept.

Mortality on any given day was assumed to follow a Poisson distribution. Time-varying confounders were controlled by indicators of day-of-week, month and year, and further stratified by whether or not the date was a public holiday. This means that temperatures on the date of death are only compared to temperatures on other days within the same calendar month, and so both known and unknown confounding factors that only change slowly over time (e.g. the age distribution of the population) are implicitly controlled for by design of the study. The matching on day-of-week and public holidays is to allow for variation in health counts by such factors, although this refinement to the model would be more important for hospitalisations data rather than for mortality. Other time-invariant factors at LAD level are also intrinsically controlled for because the mortality risks were only compared within the same LAD. However, individual-level factors cannot be controlled for. Further details of the spatial application of this model can be found in Quijal-Zamorano et al (2024).

The main effect estimate is relative risk (RR), which is the cumulative 2-week mortality risk under cold night temperatures relative to the 50^th^ percentile of winter night temperature in individual LADs. The average 50^th^ percentile night-time temperature among the LADs is 5.2 °C in England. This is in line with previous findings that although the temperature thresholds of cold-related mortality risk vary across locations, a rapid increase in cold-related mortality risks generally occurs when outdoor temperatures drop below 5-8°C (National Institute for Health and Case Excellence, 2015).

An ordinary DLNM described above requires a sufficient sample size for appropriate statistical power. Around 20 thousand deaths per location is required for a moderate effect size and statistical power (Armstrong et al, 2020). Therefore, advanced methods are needed for small area analysis with small sample sizes. In this study, the DLNM was fitted in a spatial-Bayesian framework. It assumes that the risk estimates are more similar between areas that are closer by. This is based on Tobler’s First Law of Geography— everything is related to everything else, but near things are more related than distant things (Tobler, 1970). However, in complex settings, there are also locations with sharp changes in its features resulting in discontinuities in the geographical surface, e.g. urban/rural boundaries, costal/inland boundaries (Lee & Mitchell, 2012). This is a critical limitation of this assumption and method, which can be taken into account in future research. Nevertheless, Tobler’s First Law of Geography has been used for the conceptual foundation for many classical spatial models such as inverse distance weighting, which is a practical method in modelling general spatial patterns.

By incorporating a spatial structure, the temperature-mortality association in individual LADs are fitted iteratively by borrowing results from neighbouring areas, improving the statistical power and precision of small-area analysis in LADs (Quijal-Zamorano et al, 2024). During the analysis, a neighbourhood matrix was created for pairs of LADs, where a value of 1 indicates adjacency and 0 indicates non-adjacency. The risk function for each location was fitted using data from the location itself, its adjacent locations, and the overall pooled risk function for England. This fitting process is iterative for an optimal fit for all locations. The uncertainty of temperature-mortality associations was sampled using 1000 simulations of the DLNM model. The three islands in England and Wales (Isles of Scilly, Isle of Wight, Isle of Anglesey) have been removed from the analysis because they have no neighbouring areas.

The above process is repeated for individual age groups and CoDs to obtain age and CoD-specific cold-related mortality risks. The analysis was conducted using the R dlnm and INLA package with adapted codes from Quijal-Zamorano et al (2024).

## Stage 2: Multivariate random-effects meta-regression

The coefficients of the temperature-mortality association corresponding to the median cold-related mortality risk among 1000 simulations was obtained as the central estimate from Stage 1. The variance-covariance of the coefficient was estimated from the 1000 simulations. These are the values to be pooled using the multivariate meta-regression. LADs are included as random-effects predictors of the meta-regression.

Three groups of meta-regressions were fitted with the fixed-effect predictors below:

- Meta1: deprivation + EPC
- Meta2: pension credit + EPC
- Meta3: fuel poverty

Low energy efficiency and low income are key contributors to fuel poverty. Therefore, in Meta3, fuel poverty was used as the only predictor without deprivation (or elderly deprivation) and energy efficiency. To improve model precision, the variable values are transformed into categorical values of four categorises representing the four quartiles of individual variables. For example, in the meta regressions, the variable deprivation is a categorical variable with four levels, based on the quartiles of the IMD score that the LAD corresponds to, and likewise for the EPC and fuel poverty indicators. The effect size RR was predicted using the fixed-effect predictor only to assess the modification effects of these variables on cold-related mortality risk.

The cold-related mortality risk in individual LADs were obtained by fitting the constructed meta-regression model with the actual value of the variables (e.g. actual fuel poverty level based on the quartile) and LAD-specific random-effects. Additionally, hypothetical cold-related mortality risks were also generated by predicting the meta-regression model with the lowest quartile of the fixed-effect variables for all LADs (e.g. level 1 of fuel poverty—the lowest quartile) while keeping their individual random-effects. These estimates were used to estimate the attributable fraction of cold-related mortality to fuel poverty (Meta3 model), which will be described in the next section.

The meta-regression was conducted using the R mixmeta package (Sera & Gasparrini, 2022).

## Stage 3: Population attributable burden and fraction

The population attributable burden and fraction of cold-related mortality to fuel poverty for individual age group and cause of death were quantified using the equations below.

$D_{i,d}=\left( {RR}_{t_{d}, Q_{i}}-1 \right)*P_{i}*{BMR}_{i,d}$ (Equation 1.1)

$D_{i,d}^{'}=\left( {RR}_{t_{d}, Q_{1}}-1 \right)*P_{i}*{BMR}_{i,d}$ (Equation 1.2)

${BMR}_{d}=\frac{{DMR}_{d}}{{RR}_{t_{d}}}$ (Equation 1.3)

${AD}_{i,d}=D_{i,d}-D_{i,d}^{'}=\left( {RR}_{t_{d}, Q_{i}}-{RR}_{t_{d}, Q_{1}} \right)*P_{i}*{BMR}_{i,d}$ (Equation 1.4)

${AF}_{i,d}=\frac{{AD}_{i,d}}{D_{i,d}}=\left( 1-\frac{{RR}_{t_{d}, Q_{1}}-1}{{RR}_{t_{d}, Q_{i}}-1} \right)*100\%$ (Equation 1.5)

$D_{i}=\sum_{d} D_{i,d}$ (Equation 1.6)

${AD}_{i}=\sum_{d} {AD}_{i,d}$ (Equation 1.7)

${AF}_{i}=\frac{{AD}_{i}}{D_{i}}= \frac{\sum_{d} \left( {RR}_{t_{d}, Q_{i}}-{RR}_{t_{d}, Q_{1}} \right)*P_{i}*{BMR}_{i,d}}{\sum_{d} \left( {RR}_{t_{d}, Q_{i}}-1 \right)*P_{i}*{BMR}_{i,d}}=\frac{\sum_{d} \left( {RR}_{t_{d}, Q_{i}}-{RR}_{t_{d}, Q_{1}} \right)}{\sum_{d} \left( {RR}_{t_{d}, Q_{i}}-1 \right)}$ (Equation 1.8)

The attributable cold-related deaths to fuel poverty ($AD$) for LAD $i$ on day $d$ is the difference between the actual cold-related deaths $(D_{i,d}$) (i.e. actual fuel poverty quartile) and the hypothetical cold-related deaths $D_{l,d}^{'}$ (i.e. the lowest fuel poverty quartile) from Meta 3 model. The actual deaths ($D_{i,d}$) were estimated by Equation 1.1 (Hajat et al, 2014), where RR is the relative risk of mortality at night-time temperature $t$, predicted by the meta-regression using the actual fuel poverty quartile and the LAD-specific random-effects (See stage 2). $P$ is the population in LAD $i$ in 2019. $BMR$ is the baseline daily all-cause mortality rate, which was the average monthly mortality rate (DMR) in 2015-2019 excluding deaths attributed to heat (Equation 1.3). A stationary population and DMR were assumed to isolate the contribution of temperature and fuel poverty. Hypothetical cold-related deaths associated with the lowest fuel poverty quartile ($D_{i,d}^{'}$) were estimated using ${RR}_{t_{d}, Q_{1}}$by fitting the meta-regression model with the lowest quartile of fuel poverty and the LAD-specific random-effects (Equation 1.2).

The attributable cold-related deaths to fuel poverty at LAD $i$ on day $d$ (${AD}_{i,d}$) were calculated using Equation 1.4. The attributable fraction $({AF}_{i,d}$) is the percentage of ${AD}_{i,d}$ among total cold-related deaths ($D_{i,d}$) (Equation 1.5). The number of cold-related deaths and cold-related deaths attributable to fuel poverty in on all winter days between 2007-2019 were calculated by summing $D_{i,d}$ and ${AD}_{i,d}$ across all dates with their observed temperature series (Equation 1.6 and 1.7). The attributable fraction of all cold-related mortality deaths to fuel poverty between 2007-2019 was calculated by Equation 1.8, which is not affected by the baseline mortality rate or population as shown in the equation.

The estimated $AF$ and $AD$ do not represent the burden at individual level; instead, they indicate the fraction of deaths that could have been avoided if all LADs had lower fuel poverty level (i.e. the lowest quartile of fuel poverty among all LADs). This is an underestimation of the burden of fuel poverty since even LADs in the lower quartile of fuel poverty will still include some households in fuel poverty. However, it represents a conservative, yet realistic estimation of avoidable fuel poverty-related health risks.

Monte Carlo simulations were used to estimate the empirical confidence interval (eCI) for $AF$ and $AD$. 1000 samples of the coefficients of the health risk function were generated based on the covariance matrix, assuming a multivariate normal distribution. They were combined with the temperature series between 2007-2019 to estimate the actual $AF$ in this period. The range between the 2.5^th^ and 97.5^th^ percentile of the 1000 estimates were obtained to represent the empirical confidence intervals (eCIs). This method follows the one described in Gasparrini et al (2017).

# 3. Summary of variables

Table A 1. Summary of variables including their period, source and usage.

| Variable | Description | Year/period | Dataset/ Source | Usage |
| --- | --- | --- | --- | --- |
| Mortality | Daily mortality count | Nov-Apr 2007-2019 | ONS | Stage 1 |
| Temperature | Daily night-time mean temperature (8pm previous day-8am) | Nov-Apr 2007-2019 | ECMWF ERA5-land | Stage 1 |
| Deprivation | Deprivation score | 2019 | English Index of Multiple Deprivation | Stage 2 |
| Deprivation | Percentage of household in deprivation | 2011 | Census 2011 | Stage 2 |
| Pension credit | Percentage of pensioners receiving pension credit | May 2016 & Feb 2018 | Department of Work and Pensions | Stage 2 |
| Energy performance: EPC rating | Percentage of households in EPC D and below in LADs | 2008-2024 | EPC certificates | Stage 2 |
| Energy performance: Energy consumption per floor area | Median Energy consumption per floor area of LADs | 2008-2024 | EPC certificates | Stage 2 |
| Fuel poverty | Low-Income Low-Energy Efficiency Metric | 2019 | DESNZ | Stage 2 |
| Fuel poverty | Low-Income High-Cost metric | 2018 | DESNZ | Stage 2 |
| Population | Annual population estimates | 2007-2019 | ONS | Stage 1  Stage 3 |
| Geographical boundary | Digital boundaries of LADs | 2016 | Open Geography Portal | Calculating average temperatures in individual LADs;  Result visualisation |
| Geographical boundary | Lookup table between LSOA2011 and LAD 2016 | 2011,2016 | Open Geography Portal | Data linkage |

# 4. Limitations

The average temperature in a LAD is used as the exposure to all population within the LAD. However, population may distribute unevenly within an LAD, resulting in exposure measurement error. For example, most people are concentrated in cities where the Urban Heat Island effect exists, whereas the spatial resolution of the temperature dataset (ERA5-land) is not able to fully capture it. Additionally, rural populations tend to live in valleys, which are lower altitude and thus warmer than surrounding mountains. This means that cold in rural mountainous areas may be overestimated, while temperatures in urban areas may be underestimated. Future study could use population-weighting to minimise these exposure measurement errors.

Dwelling characteristics such as flat/house and fuel type were not controlled in this study because although they may affect fuel poverty, they are less likely to affect cold-related mortality risk directly. Controlling these factors will block the casual pathways between fuel poverty and cold-related mortality risk and hence they were not controlled in this study. Additionally, local climate can affect both cold-related mortality risk (e.g. adaptation) and fuel poverty (locations with a cooler climate may be exposed to cold more and hence more prone to fuel poverty). However, climate is not controlled in the study because much of the effect of climate may operate through fuel poverty, energy efficiency and deprivation. Compared to climate, the findings on fuel poverty can support developing interventions, making the results more policy relevant.

Air pollution may affect the effect of fuel poverty on cold-related mortality risks, whereas it is not controlled in this study because although air pollution affects mortality risk, it is less likely that air pollution affects fuel poverty, and hence it is not identified as a confounder. However, there may be similar spatial distribution of air pollution and fuel poverty in some locations, and future research could take this into consideration.

Another limitation of this study is that it characterises the association between cold exposure and mortality risk over the period 2007–2019, deliberately excluding the COVID-19 pandemic period. However, the cold-related mortality risk may have shifted since 2019 due to changes in population vulnerability, health system capacity, and adaptive behaviours (Paniello-Castillo et al, 2025). This introduces uncertainty when applying the findings to future contexts, particularly in informing contemporary policy responses. While future studies should extend the analysis to include more recent years and account for the confounding impacts of COVID-19, the long historical window used in this study remains a strength. It captures a wide range of interannual variability and socioeconomic contexts, allowing for a more robust estimation of cold-related mortality risk and the modifying effects of fuel poverty, energy efficiency, and deprivation than analyses limited to more recent data alone.

# 5. Results

## Descriptive statistics

### Mortality and population

Annual winter (Nov-Apr) deaths differ hugely by LADs, mainly due to their different population sizes. The median annual winter death count across LADs in England and Wales is 195 (all-cause), 44 (CVD) and 21 (RESP) for age 0-74, and 447 (all-cause), 135 (CVD), and 77 (RESP) for age 75 and above during 2007-2019. Birmingham has the highest winter death count (age 0-74: 1611; age 75+: 2907; all-cause) between 2007-2019, and City of London has the lowest number of winter deaths (age 0-74: 3; age 75+: 9; all-cause).

The median annual winter crude mortality rate was 165 (all-cause), 38 (CVD),17 (RESP) deaths per 100,000 population among age 0-74 across the LADs, and 4122 (all-cause), 1270 (CVD), 720 (RESP) among age 75 and above. The mortality rate was generally higher in Wales and northern regions, and lower in London, South East, East of England and South West (figure A1).


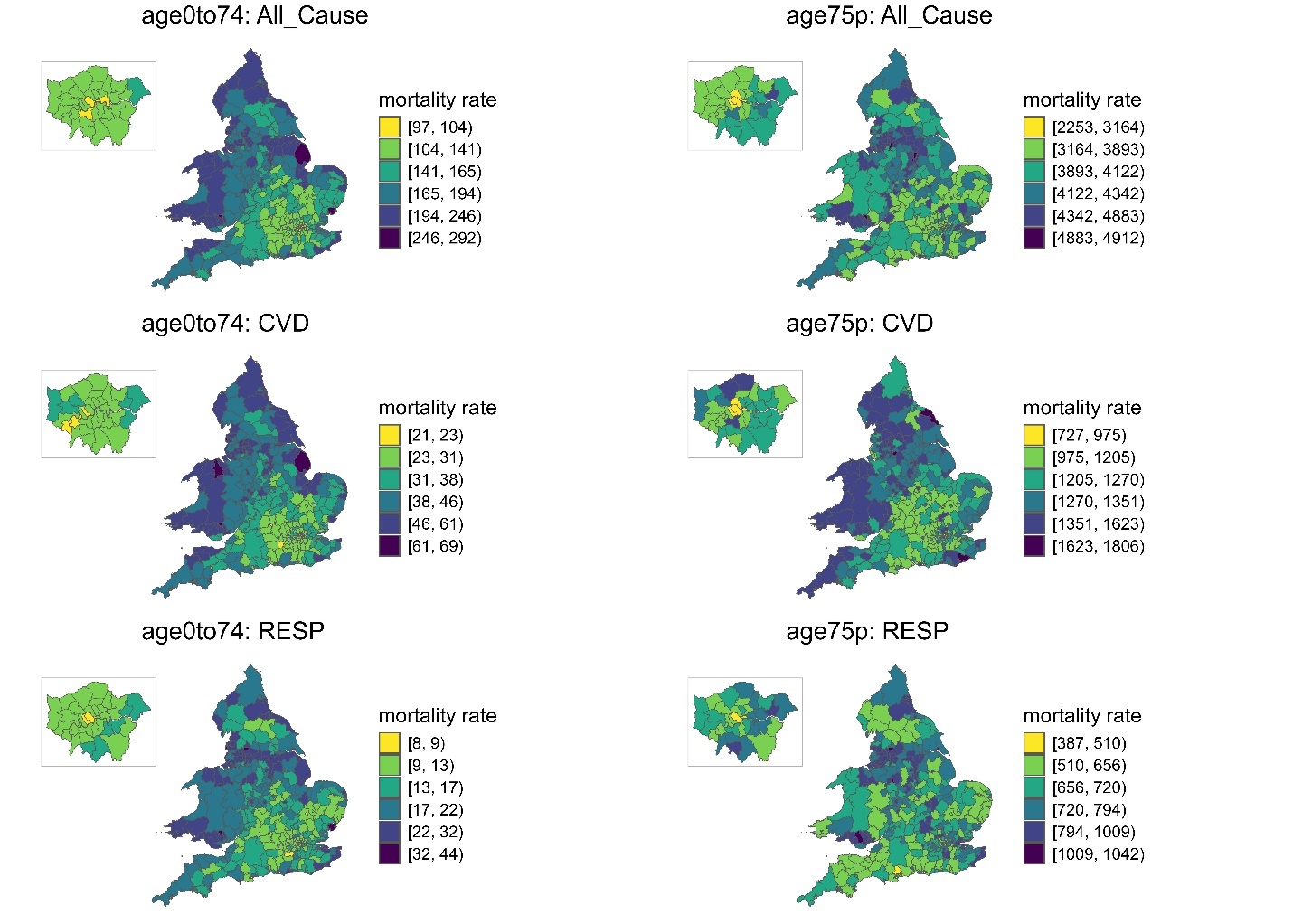


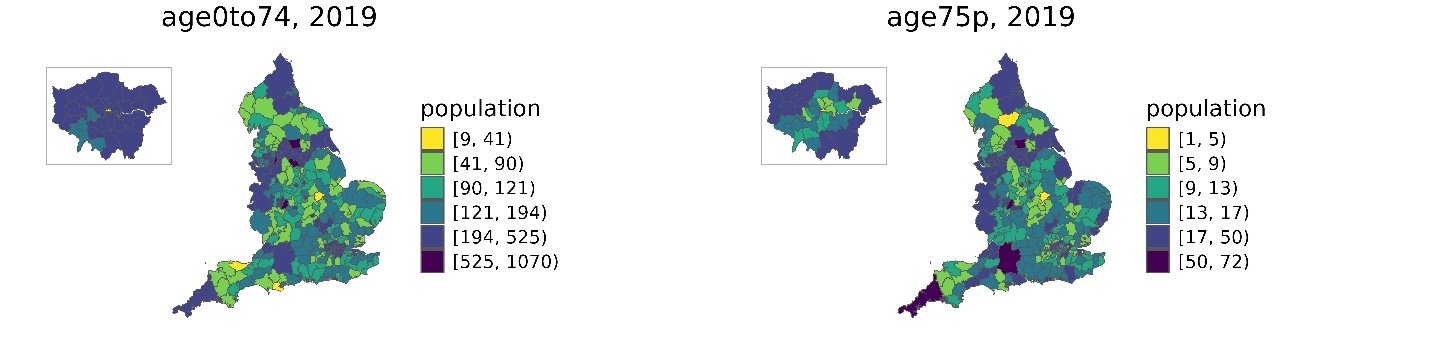


Figure A 1. Average annual winter mortality rates by age group and cause of death, and population in 2019 by age groups. This figure presents the average annual winter (Nov-Apr) mortality rate per 100,000 population for all-cause, cardiovascular (CVD), and respiratory (RESP) deaths across two age groups (0–74 and 75+) from 2007 to 2019. London is zoomed in and shown in the inset boxes. Note: The legend scale represents value ranges based on percentiles: minimum to 1st percentile, 1st to 25th, 25th to 50th, 50th to 75th, 75th to 99th, and 99th percentile to maximum. Intervals are left-inclusive and right-exclusive (e.g., [x, y)).

### Temperature

Winter night-time temperature is generally higher in coastal areas and in the south. The lowest and highest winter mean night-time temperature was in Eden (2.0 °C) (the current Westmorland and Furness unitary authority in Cumbria) and Eastbourne (7.3 °C) respectively, with a mean of 5.2°C across the LADs.

The cold risk was summarised by comparing the relative risk at the 1st, 5th and 10th percentile temperatures to the 50th percentile of local authority district-specific winter temperatures. Figure A2 illustrates these corresponding temperatures. Across all LADs the average 1st,5th,10th an 50th percentile temperatures were -3.2,-1.1,0.1 and 5.2 °C respectively.


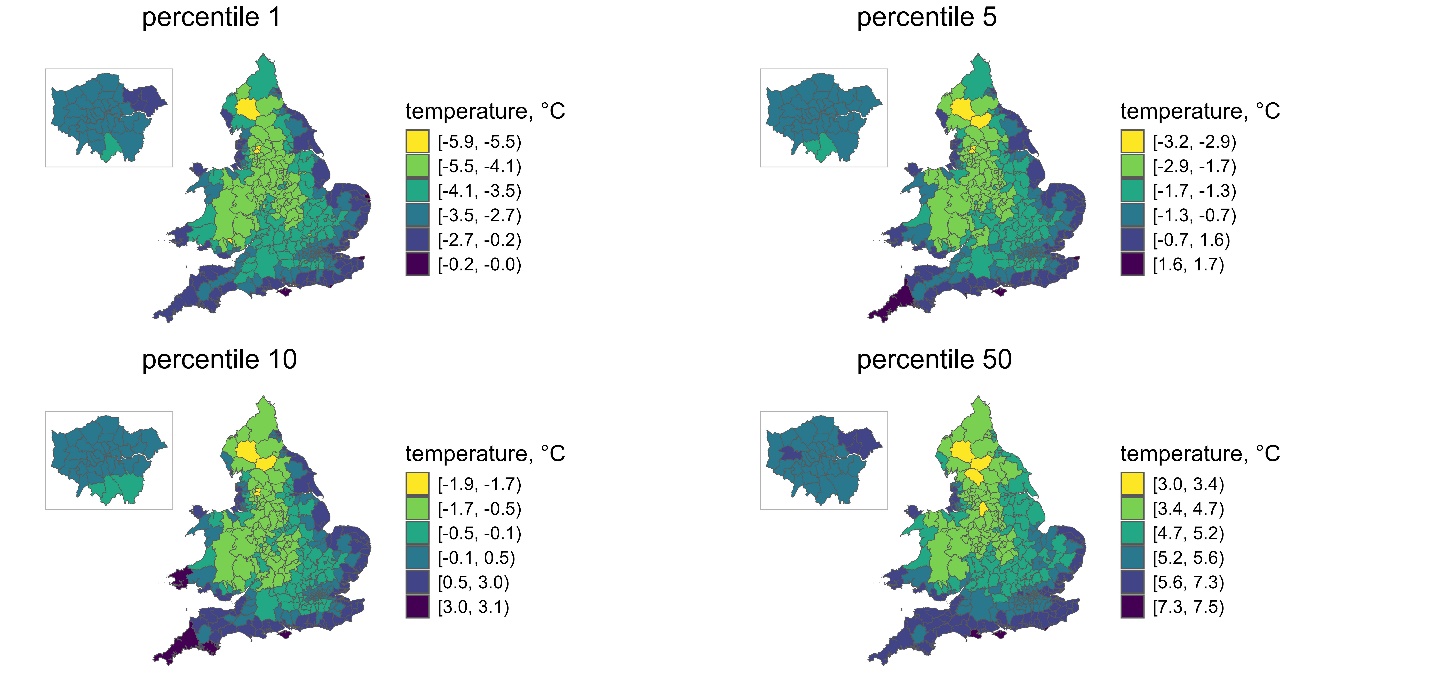


Figure A 2. Spatial distribution of daily night-time temperature percentiles (November–April, 2007–2019). This figure shows the spatial patterns of the 1^st^, 5^th^, 10^th^, and 50^th^ percentiles of daily night-time temperatures across England and Wales during the winter months (November to April) from 2007 to 2019.

### Fuel poverty, energy efficiency and deprivation

The percentage of fuel poor homes in England ranges between 2%-29% across LADs, and the range is similar across the different metrics (Figure A3). There is a high correlation between the fuel poverty measurements in England, which are above 0.7 (p<0.05) between LILEE 2019 and LIHC 2018, LIHC 2018 and LIHC 2011, and LICH 2011 and 10% income 2011 (Figure A4). There is a lower correlation between LILEE 2019 and 10% income 2011 (0.5, p<0.05).

Energy efficiency of LADs is indicated by the percentage of homes with an EPC of D or below (median: 55%, range: 24-86%) and median household annual energy consumption per floor area (median 228 kWh/m²/year, range:157-287, Figure A3). The correlation between these two metrics is very high (0.85, p<0.05, Figure A4). Therefore, this study uses EPC of D or below in the main analysis, which should be robust enough indicate energy efficiency compared to the alternative indicator due to their high correlation.

Deprivation is indicated by the IMD score (median: 19, range: 6-54) and the percentage of homes with two or more categories in deprivation from the 2011 census (median: 24%, range: 9-40%). The correlation between the two variables is extremely high (0.94, p<0.05), indicating that they are reflecting very similar features. In addition, deprivation among the older population is also indicated by the percentage of pensioners receiving pension credits (median: 7%, range: 3-22%). The correlation between pensioner deprivation and the two deprivation indicators is high (0.76, p<0.05), but it may be more sensitive to deprivation affect the older population, and hence was also used in the main analysis in this study.

Fuel poverty is positively correlated with energy efficiency, with a mean correlation of 0.5 (p<0.05) among all pairs of the four fuel poverty measures and the two energy efficiency measures. Fuel poverty is also positively correlated with deprivation, with a mean correlation of 0.5 (p<0.05) between the four fuel poverty and the two deprivation measures. The mean correlation is 0.4 (p<0.05) between fuel poverty and deprivation when including pensioner deprivation. There is also a positive correlation of 0.3 (p<0.05) (mean correlation between two energy efficiency and two deprivation measurements) between deprivation and energy efficiency, i.e. the higher the deprivation, the higher the energy efficiency. Deprived areas tends to have more social rented housing and newer built dwellings, which have an overall higher energy efficiency than owner occupied and older properties in less deprived areas (Bolton, 2024). However, the correlation is very weak, and hence particular cautious is needed in drawing on relation, particularly on household level, between home energy efficiency and deprivation.

Although with slightly different spatial distributions across different indicators, the South East region generally have fewer fuel poor, fewer deprived and more energy efficient homes (Figure A3).


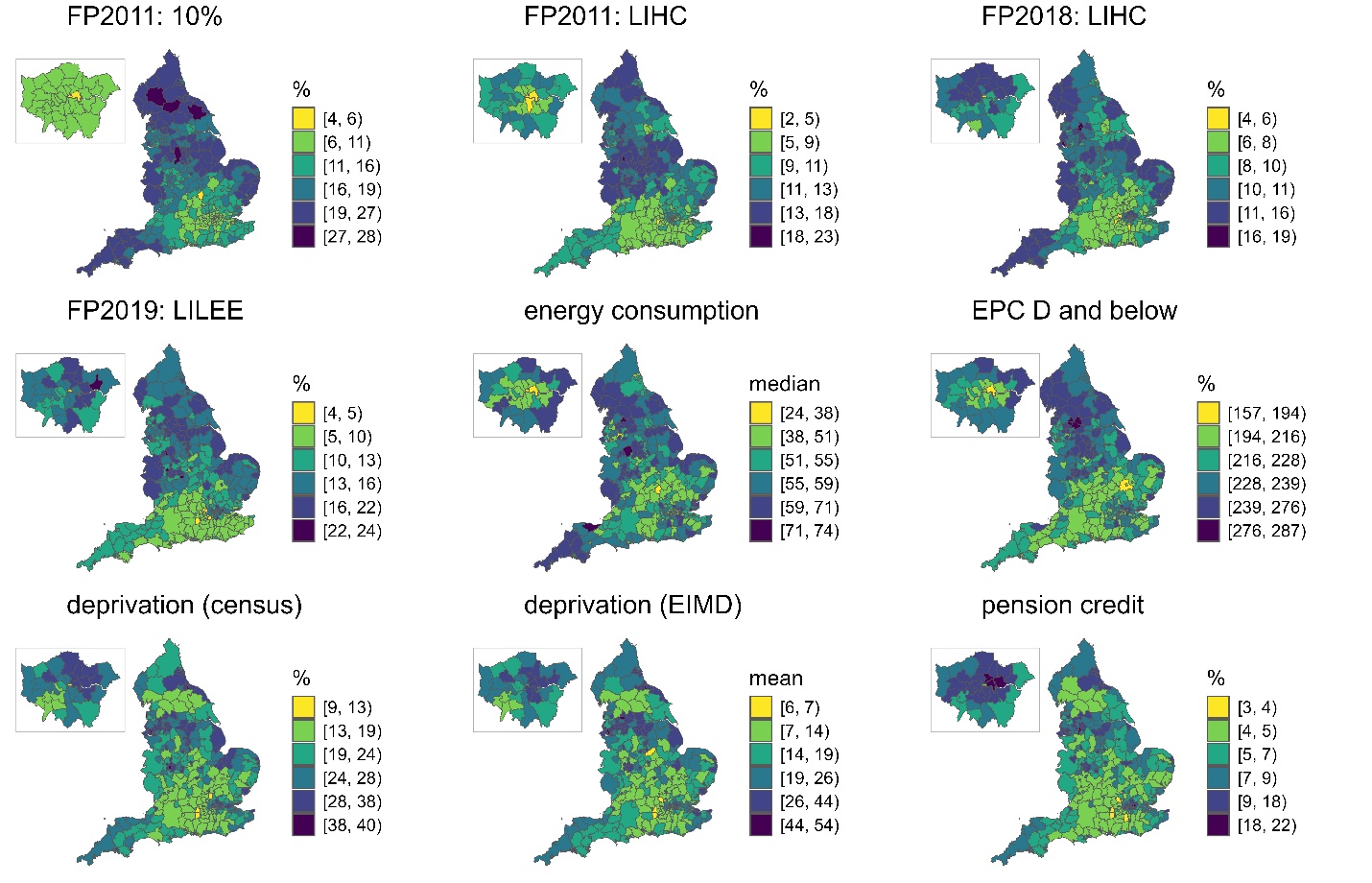


Figure A 3. Spatial distribution of fuel poverty, energy efficiency, and deprivation across Local Authority Districts in England. This figure presents four indicators of fuel poverty, two measures of energy efficiency and two indicators of deprivation and an indicator of deprivation among the older population indicated by pension credit (see Methods).


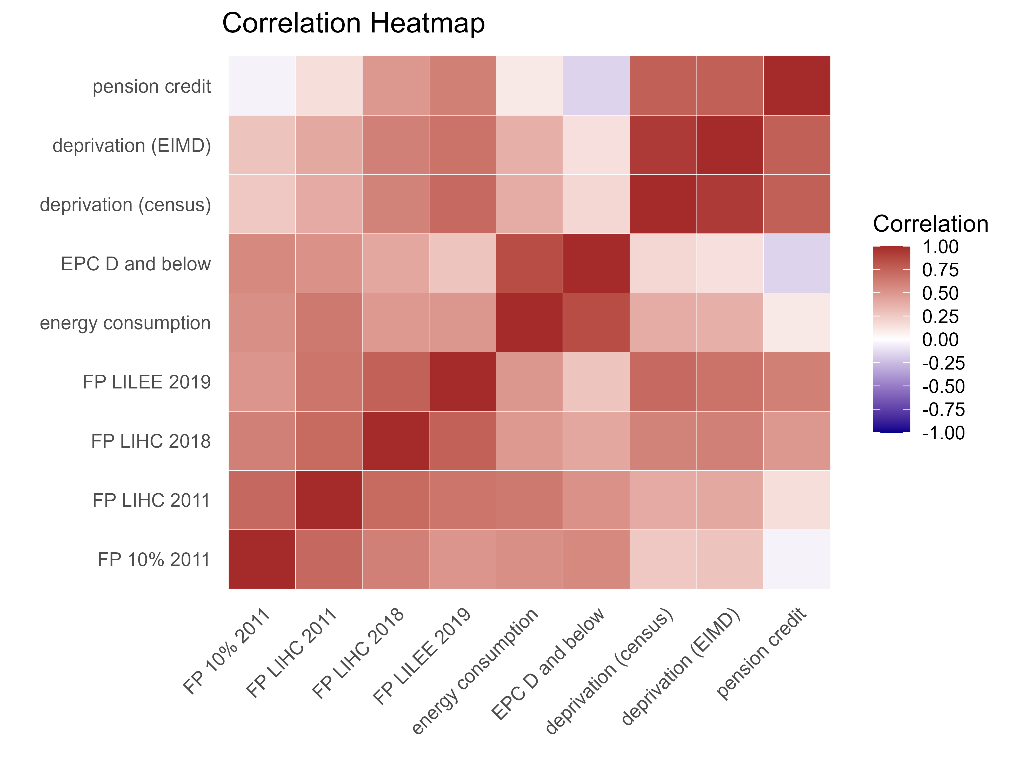


Figure A 4. Correlation between pairs of variables in England.

## Cold-related mortality risk

Summary statistics of cold-related mortality risk are provided in Table A2 and the spatial distribution across LADs is illustrated in Figure A5. The mean RR (all-cause deaths) on cold nights among all LADs is 1.12, 1.07 and 1.04 at the 1^st^, 5^th^ and 10^th^ percentile of winter night-time temperature for age 0-74, and 1.15, 1.09 and 1.06 for age 75 and above. In other words, the mortality risk increased by 12% (age 0-74) and 15% (age 75+) at the 1^st^ percentile compared to the 50^th^ percentile of winter night-time temperature.

The RR is generally higher for deaths from CVD and the highest for respiratory deaths, although with larger variation across LADs than with all-cause deaths. For age 75 and above, the mean RR is 1.22 (CVD) and 1.36 (RESP) at the 1^st^ percentile of winter night temperatures.

Table A 2. Summary statistics of relative risk at the low temperatures among the LADs. The statistics are provided for three levels of low temperatures: 1^st^, 5^th^ and 10^th^ of winter night temperature percentile, two age groups (0-74, 75+), and three causes of deaths (CoD: all cause, cardiovascular (CVD) and respiratory (RESP)). Abbreviations of the summary statistics: sd—standard deviation, min-minimum, max-maximum, Q25 and Q75—the 25^th^ and 75^th^ quantiles among the LADs.

| percentile | age | CoD | mean | median | sd | min | Q25 | Q75 | max |
| --- | --- | --- | --- | --- | --- | --- | --- | --- | --- |
| 1 | age0to74 | all cause | 1.12 | 1.12 | 0.03 | 1.03 | 1.11 | 1.14 | 1.23 |
| 5 | age0to74 | all cause | 1.07 | 1.07 | 0.02 | 1.00 | 1.06 | 1.08 | 1.13 |
| 10 | age0to74 | all cause | 1.04 | 1.04 | 0.02 | 0.98 | 1.03 | 1.05 | 1.09 |
| 1 | age75p | all cause | 1.15 | 1.15 | 0.03 | 1.07 | 1.13 | 1.17 | 1.24 |
| 1 | age75p | CVD | 1.22 | 1.23 | 0.09 | 0.96 | 1.16 | 1.28 | 1.46 |
| 1 | age75p | RESP | 1.36 | 1.36 | 0.22 | 0.85 | 1.21 | 1.48 | 3.08 |
| 5 | age75p | all cause | 1.09 | 1.09 | 0.02 | 1.03 | 1.08 | 1.10 | 1.13 |
| 5 | age75p | CVD | 1.13 | 1.13 | 0.05 | 0.93 | 1.09 | 1.16 | 1.33 |
| 5 | age75p | RESP | 1.15 | 1.14 | 0.14 | 0.87 | 1.06 | 1.23 | 2.02 |
| 10 | age75p | all cause | 1.06 | 1.06 | 0.02 | 1.01 | 1.05 | 1.07 | 1.09 |
| 10 | age75p | CVD | 1.08 | 1.08 | 0.05 | 0.91 | 1.06 | 1.11 | 1.27 |
| 10 | age75p | RESP | 1.07 | 1.06 | 0.11 | 0.81 | 0.99 | 1.14 | 1.52 |


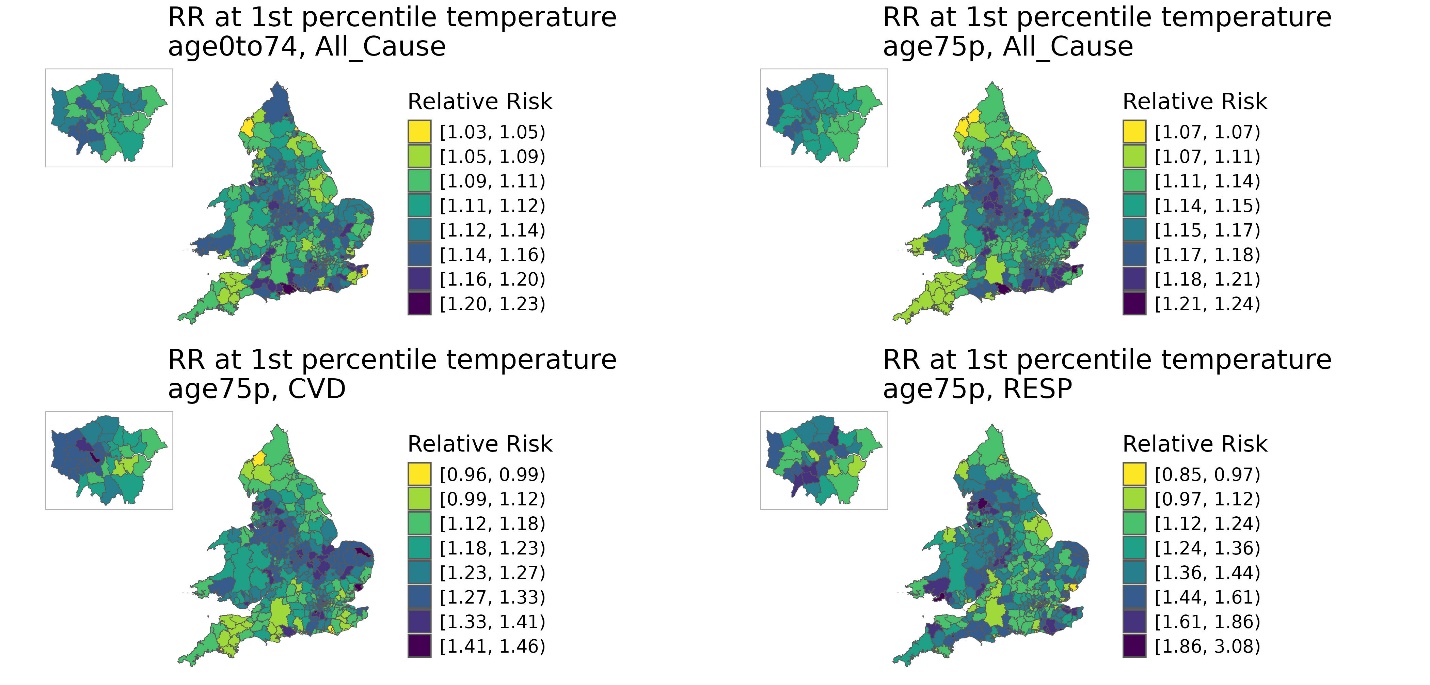


Figure A 5. Spatial distribution of cold-related mortality risk. This figure illustrates the relative risk of mortality at the 1^st^ against the 50^th^ percentile of winter night temperature distribution in individual local authority districts in England and Wales between 2007-2019 for age 0-74 from all-cause death, and age 75 plus for all-cause, cardiovascular (CVD) and respiratory deaths (RESP). RR for age 0-74 from CVD and RESP is not available due to small sample sizes. London is zoomed in and shown in the inset boxes. Note: The legend scale indicates value ranges based on percentiles: minimum to 1st percentile, 1st to 25th, 25th to 50th, 50th to 75th, 75th to 99th, and 99th percentile to maximum. Intervals are left-inclusive and right-exclusive (e.g., [x, y)).

The association between temperature and cummulative mortalty risk is given for the LAD with the most and least mortality—Birmingham and City of London respectively (Figure A6). It desmonstrates that the study power is relatively sufficient for all-cause and cardiovascular deaths even for the smallest LAD, whereas it is likely to be underpowered for respiratory deaths in very small LADs due to small sample sizes. Although the shape of the associations differ slightly by LADs, an increase in mortality risk at low temeratures was observed in all LADs.


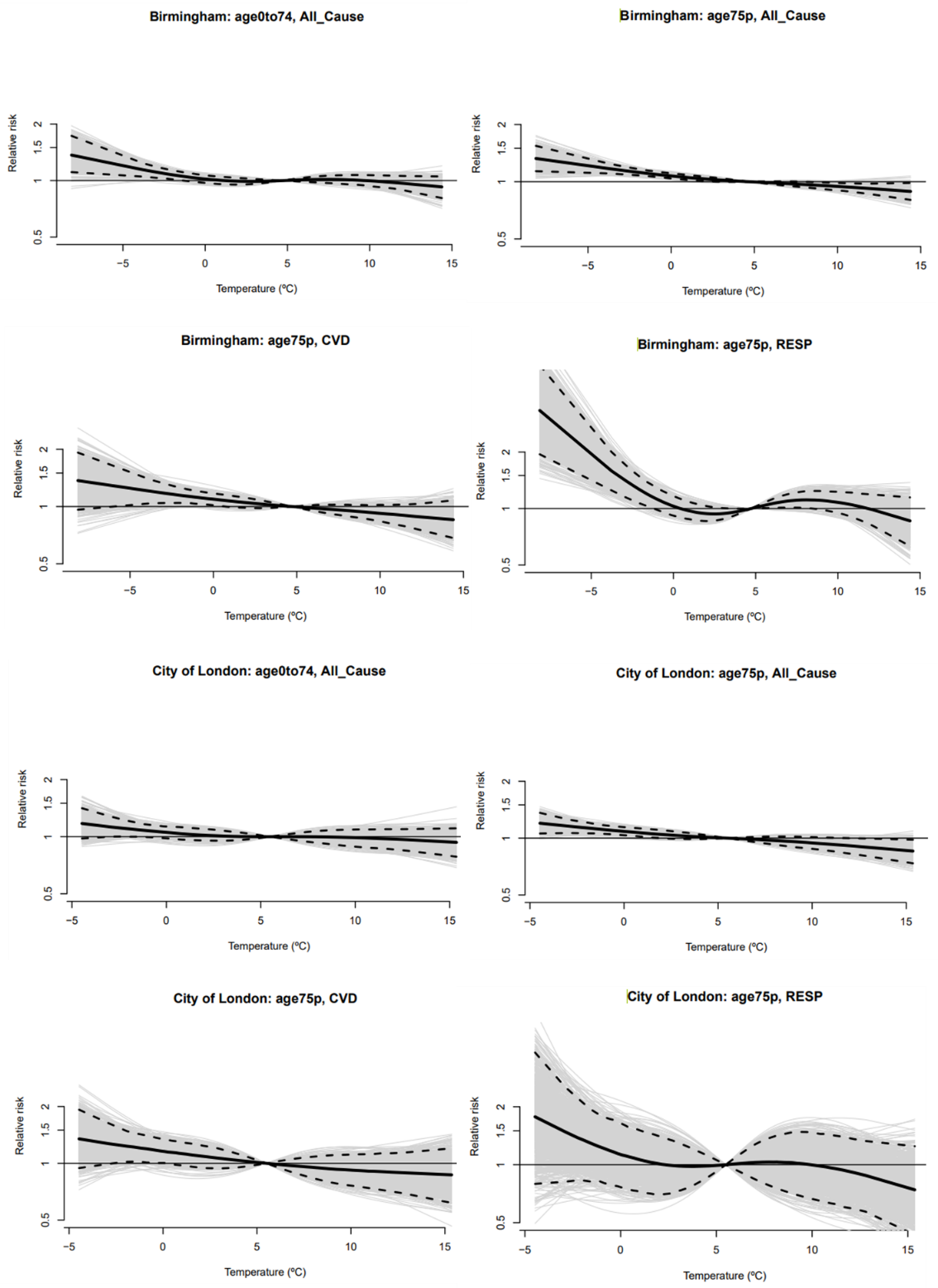


Figure A 6. Cumulative temperature–mortality associations by age group and cause of death in Birmingham and the City of London (winter 2007–2019). This figure presents the cumulative associations between winter temperature and mortality for individuals aged 0–74 (all-cause mortality), and for those aged 75 and over (all-cause, cardiovascular [CVD], and respiratory [RESP] mortality) in Birmingham and the City of London from 2007 to 2019.

## Cold effect by fuel poverty, deprivation, pension credit and energy efficiency

Table A 3. Relative risks (RR) at night-time temperatures (Temp) of –3°C and 0°C compared to 5.2°C, stratified by quartiles (Q1, Q2, Q3, Q4) of deprivation, pension credit, energy efficiency as indicated by the Energy Performance Certificate (EPC) Band D and below and fuel poverty indicator Low Income Low Energy Efficiency as measured in 2019 (FP2019: LILEE).

| age | CoD | variable | Temp | Q1 | Q2 | Q3 | Q4 |
| --- | --- | --- | --- | --- | --- | --- | --- |
| 75+ | all | deprivation | -3 | 1.139 (1.129,1.150) | 1.134 (1.124,1.145) | 1.140 (1.128,1.151) | 1.145 (1.133,1.156) |
| 75+ | all | pension credit | -3 | 1.137 (1.126,1.147) | 1.138 (1.127,1.149) | 1.140 (1.129,1.152) | 1.143 (1.132,1.155) |
| 75+ | all | EPC D and below | -3 | 1.137 (1.126,1.148) | 1.134 (1.124,1.145) | 1.139 (1.128,1.150) | 1.154 (1.143,1.166) |
| 75+ | all | FP2019: LILEE | -3 | 1.131 (1.123,1.139) | 1.144 (1.135,1.152) | 1.153 (1.144,1.161) | 1.158 (1.149,1.166) |
| 75+ | CVD | deprivation | -3 | 1.210 (1.178,1.243) | 1.201 (1.169,1.235) | 1.202 (1.168,1.238) | 1.231 (1.196,1.268) |
| 75+ | CVD | pension credit | -3 | 1.199 (1.168,1.232) | 1.209 (1.176,1.243) | 1.214 (1.179,1.250) | 1.226 (1.191,1.263) |
| 75+ | CVD | EPC D and below | -3 | 1.201 (1.168,1.235) | 1.201 (1.169,1.235) | 1.196 (1.163,1.229) | 1.243 (1.207,1.280) |
| 75+ | CVD | FP2019: LILEE | -3 | 1.158 (1.135,1.182) | 1.231 (1.205,1.258) | 1.232 (1.206,1.259) | 1.264 (1.237,1.291) |
| 75+ | RESP | deprivation | -3 | 1.206 (1.140,1.276) | 1.218 (1.151,1.288) | 1.248 (1.177,1.325) | 1.260 (1.190,1.335) |
| 75+ | RESP | pension credit | -3 | 1.192 (1.128,1.260) | 1.232 (1.163,1.305) | 1.268 (1.194,1.346) | 1.247 (1.177,1.322) |
| 75+ | RESP | EPC D and below | -3 | 1.235 (1.165,1.309) | 1.218 (1.151,1.288) | 1.351 (1.276,1.431) | 1.375 (1.294,1.461) |
| 75+ | RESP | FP2019: LILEE | -3 | 1.202 (1.152,1.254) | 1.349 (1.289,1.412) | 1.323 (1.265,1.383) | 1.372 (1.313,1.433) |
| 75+ | all | deprivation | 0 | 1.064 (1.056,1.071) | 1.063 (1.056,1.071) | 1.064 (1.057,1.072) | 1.070 (1.063,1.078) |
| 75+ | all | pension credit | 0 | 1.061 (1.054,1.069) | 1.064 (1.057,1.072) | 1.067 (1.059,1.075) | 1.070 (1.062,1.077) |
| 75+ | all | EPC D and below | 0 | 1.057 (1.050,1.065) | 1.063 (1.056,1.071) | 1.064 (1.056,1.071) | 1.062 (1.055,1.070) |
| 75+ | all | FP2019: LILEE | 0 | 1.053 (1.047,1.059) | 1.065 (1.060,1.071) | 1.065 (1.060,1.071) | 1.071 (1.066,1.077) |
| 75+ | CVD | deprivation | 0 | 1.084 (1.064,1.104) | 1.091 (1.070,1.112) | 1.089 (1.067,1.111) | 1.096 (1.075,1.117) |
| 75+ | CVD | pension credit | 0 | 1.078 (1.058,1.099) | 1.092 (1.072,1.113) | 1.094 (1.072,1.116) | 1.096 (1.075,1.118) |
| 75+ | CVD | EPC D and below | 0 | 1.085 (1.065,1.107) | 1.091 (1.070,1.112) | 1.090 (1.070,1.111) | 1.096 (1.074,1.118) |
| 75+ | CVD | FP2019: LILEE | 0 | 1.069 (1.053,1.084) | 1.096 (1.080,1.112) | 1.094 (1.078,1.110) | 1.101 (1.085,1.117) |
| 75+ | RESP | deprivation | 0 | 1.005 (0.966,1.046) | 1.029 (0.989,1.070) | 1.066 (1.024,1.109) | 1.054 (1.015,1.094) |
| 75+ | RESP | pension credit | 0 | 0.993 (0.954,1.032) | 1.038 (0.998,1.080) | 1.066 (1.024,1.110) | 1.057 (1.019,1.098) |
| 75+ | RESP | EPC D and below | 0 | 1.040 (1.000,1.083) | 1.029 (0.989,1.070) | 1.056 (1.016,1.098) | 1.099 (1.055,1.145) |
| 75+ | RESP | FP2019: LILEE | 0 | 1.001 (0.971,1.031) | 1.079 (1.047,1.112) | 1.067 (1.036,1.098) | 1.115 (1.084,1.146) |

# References

Achebak, H., Rey, G., Lloyd, S. J., Quijal-Zamorano, M., Fernando Méndez-Turrubiates, R. & Ballester, J. (2023) Drivers of the time-varying heat-cold-mortality association in Spain: A longitudinal observational study. *Environment International*, 182, 108284. doi: <https://doi.org/10.1016/j.envint.2023.108284>

Arbuthnott, K., Hajat, S., Heaviside, C. & Vardoulakis, S. (2018) What is cold-related mortality? A multi-disciplinary perspective to inform climate change impact assessments. *Environment International*, 121, 119-129. doi: 10.1016/j.envint.2018.08.053

Armstrong, B. G., Gasparrini, A., Tobias, A. & Sera, F. (2020) Sample size issues in time series regressions of counts on environmental exposures. *BMC medical research methodology*, 20(1), 15. doi: 10.1186/s12874-019-0894-6

Bolton, P. (2024) *Energy efficiency of UK homes.* House of Commons Library. Available online: <https://researchbriefings.files.parliament.uk/documents/CBP-9889/CBP-9889.pdf> [Accessed 06 Feb 2024].

Building Research Establishment (2022) *The Government’s Standard Assessment Procedure for Energy Rating of Dwellings Version 10.2 (17-03-2022).* BRE Garston, Watford. Available online: <https://files.bregroup.com/SAP/SAP%2010.2-17-03-2022.pdf> [Accessed 29 May 2025].

Department for Business, Energy & Industrial Strategy,, (2020) *Fuel Poverty Methodology Handbook (Low Income High Costs).* Available online: <https://assets.publishing.service.gov.uk/media/603fcdaee90e077dd08f15e6/Fuel_Poverty_Methodology_Handbook_2020_LIHC.pdf> [Accessed 15 Jan 2025].

Department for Levelling Up, Housing & Communities (2024) *Energy Performance of Buildings Data, England and Wales: Guidance.* Available online: <https://epc.opendatacommunities.org/docs/guidance> [Accessed 15 Jan 2025].

Gasparrini, A., Armstrong, B. & Kenward, M. G. (2010) Distributed lag non-linear models. *Statistics in Medicine*, 29(21), 2224-2234. doi: 10.1002/sim.3940

Gasparrini, A., Guo, Y., Sera, F., Vicedo-Cabrera, A. M., Huber, V., Tong, S., de Sousa Zanotti Stagliorio Coelho, M., Nascimento Saldiva, P. H., Lavigne, E., Matus Correa, P., Valdes Ortega, N., Kan, H., Osorio, S., Kyselý, J., Urban, A., Jaakkola, J. J. K., Ryti, N. R. I. & Pascal, M. (2017) Projections of temperature-related excess mortality under climate change scenarios. *The Lancet Planetary Health*, 1(9), e360-e367. doi: 10.1016/S2542-5196(17)30156-0

GOV.UK (n.d.) *Pension Credit.* Available online: <https://www.gov.uk/pension-credit/eligibility>.

Hajat, S., Chalabi, Z., Wilkinson, P., Erens, B., Jones, L. & Mays, N. (2016) Public health vulnerability to wintertime weather: time-series regression and episode analyses of national mortality and morbidity databases to inform the Cold Weather Plan for England. *Public Health*, 137, 26-34. doi: <https://doi.org/10.1016/j.puhe.2015.12.015>

Lee, D. & Mitchell, R. (2012) Boundary detection in disease mapping studies. *Biostatistics*, 13(3), 415-426. doi: 10.1093/biostatistics/kxr036

National Institute for Health and Case Excellence (2015) *Excess winter deaths and illness and the health risks associated with cold homes.* Available online: <www.nice.org.uk/guidance/ng6> [Accessed 30 Jul 2024].

ONS (2014) *2011 Census Variable and Classification Information: Part 4.* Office for National Statistics. Available online: <http://www.ons.gov.uk/ons/guide-method/census/2011/census-data/2011-census-userguide/information-by-variable/part-4--derived-variables.pdf> [Accessed 15 Jan 2025].

ONS (2023) *How fuel poverty is measured in the UK: March 2023.* Office for National Statistics. Available online: <https://www.ons.gov.uk/peoplepopulationandcommunity/housing/articles/howfuelpovertyismeasuredintheuk/march2023#comparing-the-uk-fuel-poverty-methods> [Accessed 15 Jan 2025].

ONS (2024) *Energy efficiency of housing in England and Wales: 2024.* Office for National Statistics. Available online: <https://www.ons.gov.uk/peoplepopulationandcommunity/housing/articles/energyefficiencyofhousinginenglandandwales/2024?utm_source=chatgpt.com> [Accessed 12 May 2025].

Paniello-Castillo, B., Quijal-Zamorano, M., Gallo, E., Basagaña, X. & Ballester, J. (2025) Regional changes in temperature-related mortality before and during the COVID-19 pandemic: a continental modelling analysis in 805 European regions. *Environmental Research*, 278, 121697. doi: <https://doi.org/10.1016/j.envres.2025.121697>

Quijal-Zamorano, M., Martinez-Beneito, M. A., Ballester, J. & Marí-Dell’Olmo, M. (2024) Spatial Bayesian distributed lag non-linear models (SB-DLNM) for small-area exposure-lag-response epidemiological modelling. *International Journal of Epidemiology*, 53(3). doi: 10.1093/ije/dyae061

Sera, F. & Gasparrini, A. (2022) Extended two-stage designs for environmental research. *Environmental Health*, 21(1). doi: 10.1186/s12940-022-00853-z

Tobler, W. R. (1970) A Computer Movie Simulating Urban Growth in the Detroit Region. *Economic Geography*, 46, 234-240. doi: 10.2307/143141

Wan, K., Feng, Z., Hajat, S. & Doherty, R. M. (2022) Temperature-related mortality and associated vulnerabilities: evidence from Scotland using extended time-series datasets. *Environmental Health*, 21(1). doi: 10.1186/s12940-022-00912-5
